# Prognostic Factors for Tumor Recurrence after a 12-Year, Single-Center Experience of Liver Transplantations in Patients with Hepatocellular Carcinoma

**DOI:** 10.1155/2010/904152

**Published:** 2010-08-25

**Authors:** Matteo Cescon, Matteo Ravaioli, Gian Luca Grazi, Giorgio Ercolani, Alessandro Cucchetti, Valentina Bertuzzo, Gaetano Vetrone, Massimo Del Gaudio, Marco Vivarelli, Antonietta D'Errico-Grigioni, Alessandro Dazzi, Paolo Di Gioia, Augusto Lauro, Antonio Daniele Pinna

**Affiliations:** ^1^General Surgery and Transplant Unit, Department of General Surgery and Organ Transplantation, University of Bologna, 40138 Bologna, Italy; ^2^Pathology Division, Department of Oncology and Hematology, The “Felice Addarii” Institute, University of Bologna, 40138 Bologna, Italy

## Abstract

*Background*. Factors affecting outcomes after orthotopic liver transplantation (OLT) for hepatocellular carcinoma (HCC) have been extensively studied, but some of them have only recently been discovered or reassessed. *Methods*. We analyzed classical and more recently emerging variables with a hypothetical impact on recurrence-free survival (RFS) in a single-center series of 283 patients transplanted for HCC between 1997 and 2009. *Results*. Five-year patient survival and RFS were 75% and 86%, respectively. Thirty-four (12%) patients had HCC recurrence. Elevated preoperative alpha-fetoprotein (AFP) levels, preoperative treatments of HCC, unfulfilled Milan and up-to-seven criteria at final histology, poor tumor differentiation, and tumor microvascular invasion negatively affected RFS by univariate analysis. Milan and up-to-seven criteria applied preoperatively, and the use of m-TOR inhibitors did not reach statistical significance. Cox's proportional hazard model showed that only elevated AFP levels (Odds Ratio = 2.88; 95% C.I. = 1.43–5.80; *P* = .003), preoperative tumor treatments (Odds Ratio = 4.84; 95% C.I. = 1.42–16.42; *P* = .01), and microvascular invasion (Odds Ratio = 4.82; 95% C.I. = 1.87–12.41; *P* = .001) were predictors of lower RFS. *Conclusions*. Biological aggressiveness and preoperative tumor treatment, rather than traditional and expanded dimensional criteria, conditioned the outcomes in patients transplanted for HCC.

## 1. Introduction

Hepatocellular carcinoma (HCC) is one of the five most common malignancies and its frequency is increasing in Western countries [1]. Within well-accepted criteria, liver transplantation represents the treatment of choice for patients with HCC on cirrhosis [2].

After the introduction of the so-called Milan criteria (MC), clinical outcomes after orthotopic liver transplantation (OLT) for HCC drastically improved compared to the previous era, where the inappropriate patient selection generally led to dismal results [2]. However, using MC does not completely eliminate the risk of recurrence [3, 4], while the increasing number of patients with HCC on cirrhosis with a potential benefit from OLT pushed some centers to expand classical MC, with acceptable results [5].

Cancer recurrence is greatly affected by factors different from tumor size and number, depicting a more aggressive tumor behavior, such as vascular invasion and poor differentiation [4, 6–8]. 

Recently, newly proposed dimensional criteria based on histology of the explanted native liver showed that a moderate expansion of the MC is possible, without worsening the prognosis [6]. One major criticism to these findings is that, due to the still imperfect preoperative imaging techniques, preoperative patient selection with enlarged criteria can not be safely proposed [9]. 

The impact of the posttransplant immunosuppression on tumor recurrence has recently been outlined, and some authors demonstrated a beneficial effect of Mammalian Target Of Rapamycin (m-TOR) inhibitors in the clinical setting [10, 11]. However, prospective randomized studies confirming the safer use of these drugs compared to standard immunosuppressive schedules in patients with HCC are lacking.

In the present study, we analyzed the outcomes in terms of tumor recurrence, recurrence-free survival (RFS), and disease-free survival (DFS) of a single-center series of OLTs for HCC by including traditional risk factors and those findings were recently discussed as possible determinants of prognosis, such as up-to-seven criteria [6] and the use of m-TOR inhibitors.

## 2. Material and Methods

Between January 1997 and September 2009, a total of 289 patients received orthotopic liver transplantation (OLT) at our department with a preoperative diagnosis of HCC. 

Of these 289 patients, 6 (2%) died within one month after OLT without evidence of HCC recurrence and were excluded from the analysis, while the remaining 283 (98%) formed the study population. All data were collected from a prospectively updated database. 

Preoperative diagnosis of HCC was obtained as previously reported [7]. The diagnosis of HCC was made preoperatively following the EASL and the AASLD guidelines [12, 13]. Nodules between 1 and 2 cm were considered HCCs when two radiological techniques among ultrasonography (US), computed tomography (CT), and magnetic resonance imaging (MRI) showed a typical hypervascular pattern with a washout in the venous phase. Nodules less than 2 cm with an equivocal imaging pattern were biopsied whenever possible, and, if the biopsy was technically unfeasible, they were included in a US and CT scan surveillance program. The diagnosis of HCC was the only indication for LT in 16 cases (5%), while in the remaining cases it was associated with liver failure due to cirrhosis. During the waiting time, preoperative treatments were decided in each patient by a multidisciplinary team according to the tumor stage, HCC location, and liver function. Patients with a single nodule or two nodules located in the same segment and preserved liver function were treated by liver resection. Patients not suitable for liver resection were treated by transarterial chemo-embolization (TACE), percutaneous alcohol injection (PEI), or radiofrequency ablation (RF) aiming at achieving complete tumor necrosis. Some patients were treated by different modalities over time. 

Throughout the study period, patients were included in the waiting list for OLT only if meeting the Milan criteria (MC). In selected cases, patients not meeting the MC were included in the waiting list after completing a down-staging protocol, as previously reported in [7]. A total of 39 (14%) patients were downstaged and transplanted.

The policy of our center in including/maintaining each patient in the waiting list is based on the fulfilling of the MC by considering only entirely or partially viable nodules, and not completely treated nodules, at any radiological evaluation. For example, one patient presenting with 4 nodules less than 3 cm in diameter could enter the waiting list even if, after treatment and a subsequent followup of at least 3 months, only one nodule showed complete response within the down-staging protocol. Even without being initially downstaged, patients developing multiple nodules during their history could be maintained in the waiting list if, at the more recent radiological assessment, previous lesion(s) had been successfully treated, and the still viable tumor(s) fulfilled the MC, provided that the total computation of nodules did not exceed criteria for down-staging during the entire patient surveillance [7].

However, for the purpose of the present study, MC and up-to-seven criteria were applied to all tumors (either still viable or completely treated) as detected by preoperative imaging in an intention-to-treat approach from the time of presentation to the date of transplant. Indeed, apparently necrotic lesions may display residual foci of viability at final histology. 

When listed, patients with all tumor stages were followed with US, alpha-fetoprotein (AFP) determination, and CT scan or MRI repeated at least every 3 months. 

After OLT, patients were followed-up with dosage of serum AFP level and hepatic US every 3 months, and with abdominal CT scan and chest X-ray every 6 months. In the case of suspected tumor recurrence, CT and/or MRI were performed. In selected cases, bone scan, positron emission tomography scan, angiography of the celiac trunk with Lipiodol injection, and, if necessary, liver biopsy were performed. 

Severity of liver dysfunction was graded according to the real Model for End-stage Liver Disease (MELD) score currently in use by UNOS (http://www.unos.org/) [14]. This score was retrospectively applied to patients transplanted before 2001.

Hepatitis B infection was defined as the positivity of hepatitis B surface antigen (HBsAg) or of anticore antibodies (HBcAb) at the time of surgery. Hepatitis C infection was defined as the positivity for serum anti-HCV antibodies.

### 2.1. Statistical Analysis

Results were expressed as median and range of values. Differences in tumor recurrence between groups were calculated with the with the chi-square or the Fisher exact test, when appropriate. The Kaplan-Meier method was used for the analysis of prognostic factors for RFS and DFS, and the differences between groups were compared by the log-rank test. Overall survival was computed from the day of surgery to the day of death or the last follow-up visit. RFS was computed from the day of surgery to the date of detection of tumor recurrence, and patients who died due to causes unrelated to tumor recurrence were censored at the date of death. DFS was computed from the day of surgery to the date of detection of tumor recurrence or patient death in the case of no recurrence. Variables achieving statistical significance at the univariate analysis were put in the multivariate Cox's proportional hazard model (for RFS). A *P*-value <.05 was considered statistically significant. Statistical analysis was carried out with the SPSS software packaging (SPSS Inc., Chicago, IL, USA), version 13. 

## 3. Results

### 3.1. Characteristics of the Study Population

Patient characteristics are reported in Table 1. There were 241 (85%) males and 42 (15%) females. Median age was 57 years (range: 33–68). Most patients (54%) had isolated HCV infection, while median MELD score was 15 (range: 6–45).

The large majority of patients (78%) received preoperative treatments in order to control tumor growth/invasiveness and to prevent a drop out from the waiting list. TACE was performed in 56% of patients, while PEI, RF, and liver resection were offered to a minority of subjects. 

Preoperatively, 79% and 94% of patients fulfilled the MC and the up-to-seven criteria (i.e., the sum of total number of tumors plus the diameter of the largest nodule in cm not exceeding the value of 7) [6], respectively. The above percentages changed to 73% and to 88%, respectively, at the final histology of the native liver. 

A discrepancy between preoperative and postoperative MC was found in 80 (28%) patients, with 48 (17%) patients being under-staged and 32 (11%) being over-staged. A divergence between preoperative and postoperative up-to-seven criteria was present in 34 (12%) patients, with 30 (11%) patients being under-staged and 12 (4%) being over-staged.

Poorly differentiated tumors according to the Edmonson classification [15] were found in nearly 50% of patients, while microvascular and macrovascular invasion were observed in 41% and in 1% of patients, respectively. 

Postoperatively, m-TOR inhibitors (sirolimus or everolimus) were used in 43 (15%) patients as primary immunosuppressant drugs or in combination with reduced doses of calcineurin inhibitors (tacrolimus or cyclosporine). The indications for using m-TOR inhibitors were side-effects of calcineurin inhibitors and/or histological evidence of HCC at high risk of recurrence [11]. Therapeutic levels of either sirolimus or everolimus were targeted at 3 to 8 ng/mL in all patients.

### 3.2. Survival Analysis

Median followup was 42 months (range: 1–152). At the end of followup, 214 (76%) patients were alive, and 69 (24%) had died. Causes of death were HCC recurrence in 27 (9%) cases, hepatitis recurrence in 15 (5%) cases, infections in 7 (2%) cases, multiorgan failure in 7 (2%) cases, other *de novo* tumors in 3 (1%) cases, rejection in 1 (0.4%) case, and other causes in 9 (3%) cases. Overall 3-year and 5-year patient survival rates were 81% and 75%, respectively.

Thirty-four (12%) patients had HCC recurrence at a median time from transplant of 12 months (range: 1–118). In 2 cases, HCC recurrence occurred 7 and 9 years after OLT, respectively, and it was thought to be related to posttransplant hepatitis C recurrence. 

The initial sites of tumor recurrence were liver in 3 cases, lung in 3 cases, peritoneum in 2 cases, and bone in 2 cases; multiple sites were simultaneously involved in the remaining 24 cases. Overall 3-year and 5-year RFS rates were 88% and 86%, respectively. Overall 3-year and 5-year DFS rates were 78% and 74%, respectively.

In all cases, the treatment of recurrence consisted in systemic chemotherapy with different schedules, including one patient treated with sorafenib. Combined treatments were liver resection (2 cases), radiotherapy (one case), RF (one case), and intra-arterial chemotherapy (one case). Median patient survival after recurrence was 8 months (range: 0–36).

### 3.3. Prognostic Factors for Recurrence-Free Survival and Tumor Recurrence

Patient gender and sex, preoperative AFP (with a cutoff set at 30 ng/mL, according to our previous experience) [7, 16], type of viral infection, fulfilling of MC at OLT, preoperative treatments, preoperative down-staging, fulfilling of MC at final histology of the explanted native liver, tumor differentiation, presence of micro- and macrovascular tumor invasion, and postoperative immunosuppression including m-TOR inhibitors were all analyzed for their impact on RFS. Fulfilling of the so-called up-to-seven criteria was recently shown to lead to results comparable to those obtained with the MC [6, 17]. Therefore, preoperative and postoperative up-to-seven criteria were also considered in the analysis (Table 2).

Elevated AFP levels, preoperative treatments, unfulfilled postoperative MC and up-to-seven criteria, poor tumor differentiation, and presence of microvascular invasion were all predictors of lower RFS by univariate analysis (Table 2).

Since fulfilling of postoperative MC and up-to-seven criteria were confirmed to offer similar survival rates in our series, the former variable was not put into the multivariate analysis.

Cox's proportional hazard model showed that only elevated AFP levels (Odds Ratio = 2.88; 95% C.I. = 1.43–5.80; *P* = .003), preoperative tumor treatments (Odds Ratio = 4.84; 95% C.I. = 1.42–16.42; *P* = .01), and microvascular invasion (Odds Ratio = 4.82; 95% C.I. = 1.87–12.41; *P* = .001) were predictors of lower RFS (Table 3). 

The same above reported variables were investigated as putative predictive factors for tumor recurrence within one year from transplant (Table 4). Elevated preoperative AFP levels and preoperative tumor treatment were correlated with higher one-year recurrence rates.

Three- and 5-year overall survival and RFS rates of 48 patients understaged with regard to preoperative MC were 73% and 67% and 79% and 76%, respectively.

Three- and 5-year overall survival and RFS rates of 30 patients understaged with regard to preoperative up-to-seven criteria were 62% and 58% and 68% and 64%, respectively.

### 3.4. Effect of Down-Staging, Milan Criteria, and Up-To-Seven Criteria

Regarding patients included in the down-staging protocol, 3- and 5-year RFS of 21 patients fulfilling MC at final histology was significantly better than RFS of 18 patients not fulfilling MC (95% and 95% versus 69% and 62%, resp.; *P* = .02).

Three- and 5-year DFS rates of patients receiving dowstaging, of patients fulfilling preoperative MC and receiving preoperative treatments, and of patients within preoperative MC and receiving no preoperative treatment were 76% and 64%, 76% and 74%, and 84% and 82%, respectively (*P* = .3). By considering only patients fulfilling preoperative MC, those receiving preoperative treatments showed 3- and 5-year DFS rates comparable to those receiving no treatment (77% and 72% versus 84% and 82%, resp.; *P* = .2). 

**Figure 1:**
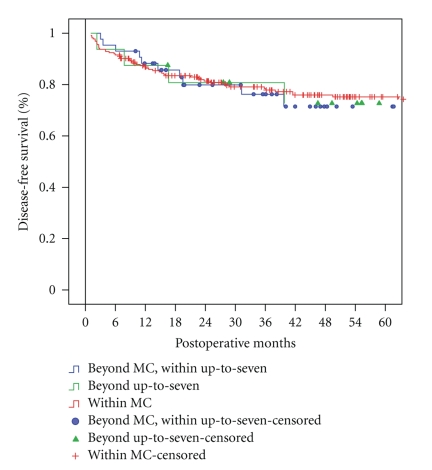
Disease-free survival of patients within preoperative MC (*n* = 224), beyond preoperative MC but within up-to-seven criteria (*n* = 43), and beyond preoperative up-to seven criteria (*n* = 16) (*P* = .8).

Three- and 5-year DFS of patients within preoperative MC (*n* = 224), beyond preoperative MC but within up-to-seven criteria (*n* = 43), and beyond preoperative up-to seven criteria (*n* = 16) were 78% and 75%, 76% and 71%, and 81% and 73%, respectively (*P* = .8) (Figure 1).

**Figure 2:**
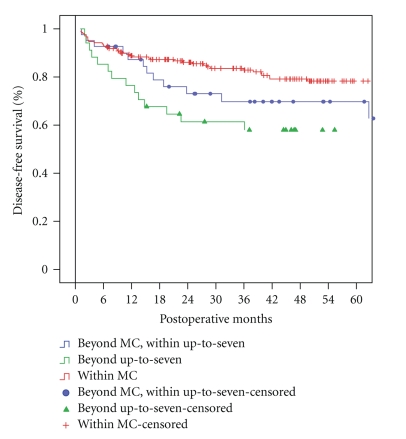
Disease-free survival of patients within histological MC (*n* = 208), beyond histological MC but within up-to-seven criteria (*n* = 41), and beyond histological up-to seven criteria (*n* = 34) (*P* = .02).

Three- and 5-year DFS of patients within histological MC (*n* = 208), beyond histological MC but within up-to-seven criteria (*n* = 41), and beyond histological up-to seven criteria (*n* = 34) were 83% and 78%, 70% and 70%, and 61% and 58%, respectively (*P* = .02) (Figure 2). Preoperative treatments were equally distributed among these latter categories of patients, being performed in 164 (81%), 32 (82%), and 26 (79%) patients, respectively (*P* = .9).

## 4. Discussion

In the present single-center series of OLT for HCC covering 12 years of activity, we took into consideration several variables with a hypothetical impact on tumor recurrence. Some of these factors, such as new dimensional criteria based on postoperative histology and extending the MC, and the type of posttransplant immunosuppression, have been recently reported [6, 11]. However, to our knowledge there are no other studies considering the classical and the more recently proposed variables altogether.

Our series confirmed the still relevant discrepancy between criteria based on imaging and on final histology, with a substantial proportion of patients being under- or over-staged before OLT. For over-staged cases, most of the inconsistencies were ascribable to the adoption of a down-staging protocol [7], but an important proportion of patients finally revealed under-staged.

As far as a drastic improvement of radiological imaging will not be achieved, preoperative and postoperative tumor morphologies will remain distinct entities, and dimensional criteria will not be completely reliable, especially when attempting to extend traditional criteria for OLT. 

The up-to-seven threshold proved accurate in anticipating the prognosis when applied postoperatively, as demonstrated by the different outcomes compared to those of patients within histological MC or beyond MC but within up-to-seven. However, this novel categorization would have a limited value if applied to the selection of ideal candidates to OLT [9].

Contrary to our previous observation [16], fulfilling of preoperative MC was not predictive of RFS. Even if with a low number of patients outside the up-to-seven criteria applied preoperatively, neither of these factors did reach statistical significance at the univariate analysis for RFS. 

In general, tumor biology and degree of invasiveness, as expressed by preoperative AFP values and microvascular invasion, rather than dimensional parameters showed a relevant impact on HCC recurrence. In particular, AFP was the only preoperative variable with an independent impact on RFS. Many different cutoffs of preoperative AFP have been reported, ranging from 10 to 1000 ng/mL [5, 18–20]. We set our cutoff at 30 ng/mL based on our previous experience showing optimal discrimination capacity [7, 16] and on the low median level (9 ng/mL) of our study population, which suggested not to chose higher threshold values.

On the other hand, microvascular invasion was present in a very high proportion of our patients and played a critical role in worsening the outcomes.

Interestingly, preoperative treatments administered with the purpose of limiting tumor invasiveness led to lower survival rates. These procedures were extensively applied, often in combination with each other, either in patients undergoing down-staging or in those developing multiple nodules during waiting time; both categories of candidates are more likely to display tumors exceeding conventional criteria at histology. 

A more limited use of any preoperative treatment in patients never exceeding preoperative MC has been reported by our group [7], suggesting that locoregional or surgical procedures were more often performed in unfavorable cases with aggressive tumor behavior, while small, single, and/or slowly growing cancers were frequently left untreated. 

However, when looking at 3 different risk categories at final histology, such as patients within MC, those beyond MC and within up-to-seven criteria, and those beyond up-to-seven criteria, we found that pretransplant treatments were equally distributed, supporting an independent role of neoadjuvant procedures in determining the outcomes. One possible explanation could be the increased tendency to invasiveness caused by a partial tumor destruction; in fact, partial necrosis achieved after TACE, PEI, or RF may enhance tumor spread [21], and this phenomenon has in turn a higher chance to be observed in larger or multiple nodules. 

Given the acceptable overall results of OLT for HCC at our center, it is our policy to maximize any tumor treatment, even with surgical excision (whenever feasible), due to long waiting periods on list expected for many patients [22]. In this view, an accurate estimation of the balance between the harm caused by liver resection in cirrhotic patients and the benefit offered to patients listed for OLT through a strategy of primary resection and salvage transplantation has recently been reported [23].

The role of immunosuppression, and particularly the use of m-TOR inhibitors with the aim of preventing tumor recurrence due to the antineoplastic properties of these drugs, is not yet fully clarified.

Our analysis could not confirm the beneficial effect of sirolimus on the recurrence of HCC shown by recent reports, including one from our institution [10, 11]. However, the study from Vivarelli et al. [11] was a case-control one, with a particular reference to patients with HCC at a higher risk of recurrence, that is, those with one or more poor prognostic factors. It is likely that the positive effect of m-TOR inhibitors becomes evident in cases of cancers with high tendency to recur.

## 5. Conclusions

We evaluated the results of our 12-year experience of OLT for HCC by considering traditional and more recently proposed prognostic factors for tumor recurrence. In particular, up-to-seven criteria proved effective in predicting the outcomes when applied postoperatively, but they showed a negligible importance if used preoperatively. Biological factors are the most important determinants of survival, and among them preoperative treatments may play a critical, possibly negative, role.

## Figures and Tables

**Table 1:** Demographic, clinical, and pathological parameters of 283 patients with HCC receiving OLT at the Bologna Center between 1997 and 2009.

Variables	
Gender	
Male	241 (85%)
Female	42 (15%)
Median age (years)	57 (33–68)

Viral infection	
HCV-positive	152 (54%)
HBV-positive	70 (25%)
HCV, HBV-positive	13 (5%)
HCV, HBV-negative	48 (17%)
Median real MELD score	15 (6–45)

Preoperative treatments	222 (78%)
TACE	158 (56%)
PEI	30 (10%)
RF	63 (22%)
Liver resection	16 (6%)

Median AFP level at OLT (ng/mL)	9 (1–6826)
Median preoperative tumor number	2 (1–6)
Median preoperative tumor diameter (cm)	2.5 (0.8–5)
Down-staging to MC	39 (14%)
Fulfilling MC at OLT	224 (79%)
Fulfilling up-to-7 criteria at OLT	267 (94%)
Median tumor number at histology	1 (0–12)
Median tumor diameter at histology (cm)	2.5 (0–6)
Fulfilling MC at histology	208 (73%)
Fulfilling up-to-7 criteria at histology	249 (88%)

Tumor grading	
G0–G2	143 (50.5%)
G3–G4	140 (49.5%)

Microvascular invasion	117 (41%)
Macrovascular invasion	3 (1%)
Post-OLT M-TOR inhibitors	43 (15%)

HCC: hepatocellular carcinoma. OLT: orthotopic liver transplantation. HCV: hepatitis C virus. HBV: hepatitis B virus. MELD: Model for End-stage Liver Disease. TACE: trans-arterial chemoembolization. PEI: percutaneous alcohol injection. RF: radiofrequency ablation. AFP: alpha-fetoprotein. MC: Milan criteria.

**Table 2:** Univariate analysis of factors affecting recurrence-free survival after OLT in patients with HCC.

Variables		3-yr survival	5-yr survival	*P* value
Gender	Female (*n* = 42)	82%	82%	.1
	Male (*n* = 241)	89%	87%	

Age	≤ 60 years (*n* = 184)	88%	87%	.9
	> 60 years (*n* = 99)	89%	85%	

AFP > 30 ng/mL at OLT	No (*n* = 201)	92%	91%	<**.0001**
	Yes (*n* = 78)	76%	74%	

Viral infection	HCV-positive (*n* = 152)	85%	83%	.4
	HBV-positive (*n* = 70)	88%	88%	
	HCV, HBV-positive (*n* = 13)	92%	92%	
	HCV, HBV-negative (*n* = 48)	98%	94%	

Down-staging to MC	No (*n* = 243)	89%	88%	.2
	Yes (*n* = 39)	83%	79%	

Preoperative MC	Fulfilled (*n* = 224)	90%	89%	.07
	Not fulfilled (*n* = 59)	81%	77%	

Preoperative up-to-seven	Fulfilled (*n* = 267)	88%	86%	.9
	Not fulfilled (*n* = 16)	86%	86%	

Pre-OLT treatments	No (*n* = 53)	96%	94%	**.05**
	Yes (*n* = 222)	86%	84%	

Histological MC	Fulfilled (*n* = 208)	94%	92%	<**.0001**
	Not fulfilled (*n* = 75)	73%	71%	

Histological up-to-seven	Fulfilled (*n* = 249)	91%	89%	<**.0001**
	Not fulfilled (*n* = 34)	69%	65%	

Grading	G0–G2 (*n* = 143)	96%	95%	<**.0001**
	G3–G4 (*n* = 140)	80%	78%	

Microvascular invasion	No (*n* = 166)	97%	96%	<**.0001**
	Yes (*n* = 117)	76%	73%	

Macrovascular invasion	No (*n* = 280)	88%	87%	.07
	Yes (*n* = 3)	67%	67%	

Post-OLT m-TOR inhibitors	No (*n* = 240)	89%	87%	.2
	Yes (*n* = 43)	81%	81%	

OLT: orthotopic liver transplantation. HCC: hepatocellular carcinoma. AFP: alpha-fetoprotein. MC: Milan criteria. M-TOR**: **Mammalian Target Of Rapamycin.

**Table 3:** Multivariate analysis of factors affecting recurrence-free survival after OLT in patients with HCC.

	*P* value	Odds Ratio	C.I. 95%
AFP > 30 ng/mL at OLT	**.003**	2.88	1.43–5.80
Pre-OLT treatments	**.01**	4.84	1.42–16.42
Histological up-to-7	.7	0.84	0.32–2.18
Grading	.4	1.43	0.56–3.65
Microvascular invasion	**.001**	4.82	1.87–12.41

OLT: orthotopic liver transplantation. HCC: hepatocellular carcinoma. AFP: alpha-fetoprotein.

**Table 4:** Analysis of factors affecting one-year tumor recurrence rate in patients with HCC.

Variables		One-yr recurrence *n* (%)	*P* value

Gender	Female (*n* = 42)	10 (24)	.3
	Male (*n* = 241)	43 (18)	

Age	≤ 60 years (*n* = 184)	34 (18)	.8
	> 60 years (*n* = 99)	19 (19)	

AFP > 30 ng/mL at OLT	No (*n* = 201)	30 (15)	**.01**
	Yes (*n* = 78)	22 (28)	

Viral infection	HCV-positive (*n* = 152)	30 (20)	.8
	HBV-positive (*n* = 70)	13 (19)	
	HCV, HBV-positive (*n* = 13)	3 (23)	
	HCV, HBV-negative (*n* = 48)	7 (15)	

Down-staging to MC	No (*n* = 243)	46 (19)	.8
	Yes (*n* = 39)	7 (18)	

Preoperative MC	Fulfilled (*n* = 224)	44 (20)	.4
	Not fulfilled (*n* = 59)	9 (15)	

Preoperative up-to-seven	Fulfilled (*n* = 267)	51 (19)	.7
	Not fulfilled (*n* = 16)	2 (12)	

Pre-OLT treatments	No (*n* = 53)	4 (7)	**.02**
	Yes (*n* = 222)	47 (21)	

Histological MC	Fulfilled (*n* = 208)	36 (17)	.3
	Not fulfilled (*n* = 75)	17 (23)	

Histological up-to-seven	Fulfilled (*n* = 249)	45 (18)	.4
	Not fulfilled (*n* = 34)	8 (23)	

Grading	G0–G2 (*n* = 143)	23 (16)	.2
	G3–G4 (*n* = 140)	30 (21)	

Microvascular invasion	No (*n* = 166)	25 (15)	.06
	Yes (*n* = 117)	28 (24)	

Macrovascular invasion	No (*n* = 280)	52 (19)	.4
	Yes (*n* = 3)	1 (33)	

Post-OLT m-TOR inhibitors	No (*n* = 240)	47 (20)	.3
	Yes (*n* = 43)	6 (14)	

OLT: orthotopic liver transplantation. HCC: hepatocellular carcinoma. AFP: alpha-fetoprotein. MC: Milan criteria. M-TOR**: **Mammalian Target Of Rapamycin.
